# Synthetic upcycling of polyacrylates through organocatalyzed post-polymerization modification†
†Electronic supplementary information (ESI) available. See DOI: 10.1039/c7sc02574b


**DOI:** 10.1039/c7sc02574b

**Published:** 2017-09-29

**Authors:** Charles P. Easterling, Tomohiro Kubo, Zachary M. Orr, Gail E. Fanucci, Brent S. Sumerlin

**Affiliations:** a George & Josephine Butler Polymer Research Laboratory , Center for Macromolecular Science & Engineering , Department of Chemistry , University of Florida , PO Box 117200 , Gainesville , FL 32611-7200 , USA . Email: fanucci@chem.ufl.edu ; Email: sumerlin@chem.ufl.edu ; Fax: +1 352 392 9741

## Abstract

The direct transformation of commercially available commodity polyacrylates into value-added materials was achieved. We demonstrate how 1,5,7-triazabicyclo[4.4.0]dec-5-ene, serving as a nucleophilic catalyst, can be used to catalyze acyl substitution reactions of acrylic polymers in the presence of alcohol and amine nucleophiles. Furthermore, we found that organocatalytic transesterification exhibits high selectivity towards sterically unhindered esters, thus providing a new route towards site-selective acyl substitution of macromolecular materials. Combining this methodology with reversible-deactivation radical polymerization (RDRP) techniques such as reversible addition–fragmentation chain-transfer (RAFT) polymerization allowed for the precise functionalization of sterically-differentiated acrylic copolymers and polymeric chain ends. We envision this approach to expedite functional polymer synthesis and provide access to functional macromolecules prepared from inexpensive, hydrolytically-stable polymeric precursors.

## Abstract 2

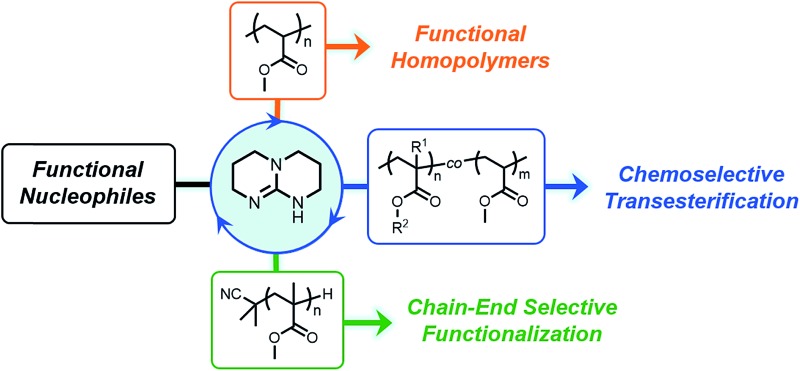
A new method for converting commodity polymeric materials into value-added specialty materials was developed *via* site-selective and chemoselective transesterification of block copolymers, statistical copolymers, and homopolymers.

## Introduction

Polymers containing activated esters have received significant advances in recent decades and have allowed for facile access to highly functional macromolecules *via* post-polymerization modification.^1^–^9^ These precursor polymers, which may serve as modular scaffolds for a plethora of subsequent functionalizations,^5^,^10^ are currently prepared from electron deficient monomers (*e.g.*, pentafluorophenyl acrylate) that are capable of undergoing amidation and transesterification.^11^ Although these materials exhibit good solubility in a variety of organic solvents, they are vulnerable to hydrolysis by moisture and trace water due to their electron-deficient nature, thus preventing their use on large scales and their storage under non-stringent conditions.

To circumvent this issue, we reasoned that an activation strategy through organocatalysis could prove useful, wherein the activation of stable esters on commodity polymers could be readily achieved through chemical or physical means; however, this activation approach has been severely underdeveloped.^12^ One alternative pathway towards the activation of esters is through the use of chemical catalysis. In contrast to acylation reactions mediated by activated esters, catalytic ester activation exhibits high chemoselectivity^13^ and provides a greener, more sustainable route towards the synthesis of esters and amides due to the increased atom economy. The application of organocatalysis in synthetic polymer chemistry has become indispensable in the synthesis of polyesters, and more recently, polyethers and polyphosphoesters through ring-opening polymerization techniques.^14^–^16^ Organocatalysts such as N-heterocyclic carbenes, 1,8-diazabicyclo[5.4.0]undec-7-ene, and 1,5,7-triazabicyclo[4.4.0]undec-5-ene (TBD) are known to efficiently catalyze transesterification and amidation reactions,^17^ even on pilot-plant scales;^18^ however, few reports have utilized the high activity of these catalysts in the synthesis of functional materials during post-polymerization modification.^19^

The direct catalytic activation and subsequent functionalization of macromolecules prepared from inexpensive commercially-relevant monomers would facilitate rapid material development and could provide a means of upcycling consumer waste material.^20^ Such examples of polymer upcycling for post-polymerization modification include the functionalization of polybutadiene,^21^ poly(methyl methacrylate) (PMMA),^22^ as well as polyolefins.^23^ Polyacrylates such as poly(methyl acrylate) (PMA) have recently undergone efficient metal-catalyzed transesterification^24^ in the synthesis of functional materials; however, the use of hydrolytically-unstable transition metal catalyst limits the potential applicability.^25^ Recently, Sawamoto reported a titanium alkoxide catalyst capable of transesterifying poly(methyl methacrylate) (PMMA) in a site-selective fashion to synthesize “pin-point functionalized” PMMA^26^ as well as gradient copolymers.^27^ This elegant approach relied on steric differentiation of methyl esters along the backbone and at the end groups of chlorine-terminated PMMA prepared by atom transfer radical polymerization. To our knowledge, this is the only known method of chemoselective transesterification of acrylic polymers without the need for activated esters.

Herein, we demonstrate the efficient functionalization of acrylic polymers and copolymers that allows for quantitative substitution of polymer side chains using TBD as a nucleophilic organocatalyst. Furthermore, chain-end selective transesterification of PMMA was achieved with the absence of competing side-chain transesterification, highlighting the high selectivity of TBD-catalyzed transesterification.

## Results and discussion

### Organocatalyzed transesterification of poly(methyl acrylate)

We reasoned that functional polymers could be directly synthesized from hydrolytically-stable, commercially-available, commodity polymers *via* organocatalysis. The approach we report provides a new straightforward method of chemoselectively and efficiently modifying vinyl polymers derived from acrylates and methacrylates. We envision this chemistry, which relies on a platform suitable to large-scale synthetic modification, will streamline access to functional materials derived from polyacrylates.

This study began by synthesizing PMA by reversible addition–fragmentation chain transfer (RAFT) polymerization^28^ with the intention of the resulting polymer serving as a substrate for acyl substitution reactions catalyzed by TBD, a nucleophilic organocatalyst. Aminolysis of the terminal trithiocarbonate followed by subsequent thio-Michael addition end-capping with methyl acrylate yielded the PMA homopolymer (*M*_n GPC MALS_ = 9800 g mol^–1^, *Đ* = 1.2) (Fig. S1†).

When PMA was subjected to 30 mol% TBD in the presence of benzyl alcohol (BnOH, 4 equiv.) at 80 °C in a closed reaction flask, incomplete conversion was observed, likely due to competitive transesterification of benzyl alcohol and the methanol produced over the course of the reaction.

**Fig. 1:**
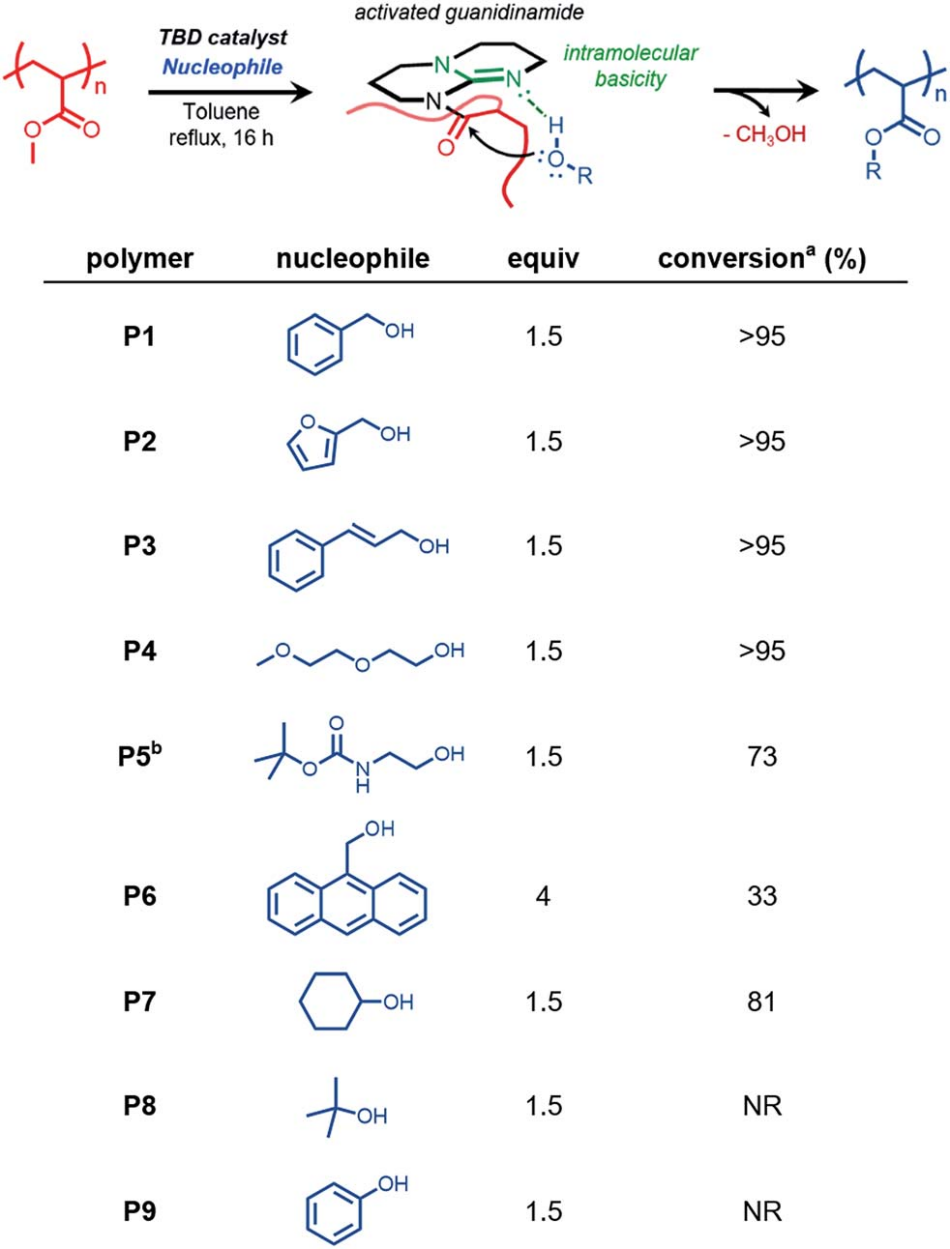
Transesterification of poly(methyl acrylate) (PMA) with various alcohol nucleophiles. PMA concentration of 100 mg mL^–1^, [TBD] = 15 mol% relative to methyl acrylate repeat units, NR: no reaction, ^a^conversion determined using ^1^H NMR spectroscopy. ^b^performed at 80 °C.

However, when the reaction conditions were optimized to allow for continuous removal of the methanol byproduct by continuous argon purging, quantitative conversion was achieved within 16 h. (Fig. 1, polymer **P1**), as confirmed by ^1^H nuclear magnetic resonance (NMR) spectroscopy by the appearance of benzylic methylene protons exhibiting a chemical shift (*δ*) at 5 ppm (Fig. S2†).

We next sought to introduce functional alcohols capable of downstream functionalization. Both resultant polymers **P2** and **P3**, containing furfuryl and olefin functionality respectively, were prepared *via* quantitative functionalization, verifying the compatibility of these reaction conditions to functional groups that could be subsequently used in Diels–Alder and thiol–ene transformations. Additionally, the preparation of a thermoresponsive polymer (**P4**) was carried out using diethylene glycol methyl ether. We then studied the compatibility of TBD-catalyzed transesterification in the presence of *tert*-butyloxycarbonyl (boc) protecting groups. Transesterification reactions with *N*-boc-ethanolamine (**P5**) led to boc deprotection and subsequent gelation under reflux conditions in toluene; however, when the reaction was conducted at 80 °C, conversion of *ca.* 73% was observed, suggesting this approach is compatible with urethane functional groups and protected amines. 9-Anthracenemethanol however showed low incorporation (*ca.* 33%) into PMA, even at a higher stoichiometric ratios. This reduced efficiency is likely attributed to steric effects of the anthracenyl groups preventing subsequent nucleophilic attack of neighboring methyl esters once appended to the polymer chain.

Next, we further studied the effect of sterics on this methodology by choosing sterically hindered alcohols such as cyclohexanol and *tert*-butanol as model nucleophiles. Cyclohexanol appeared to undergo efficient functionalization (*ca*. 81%), although giving lower conversion due to sterics. In the case of *tert*-butanol, no transesterification product was observed. Lastly, we wanted to study phenol as a nucleophile capable of acyl substitution. We anticipated hydroxyl nucleophiles with low p*K*_a_ values would exhibit efficient transesterification kinetics due to the general base activation mechanism between TBD and the approaching nucleophile.^17^ However, under these reaction conditions, no conversion was observed, likely due to catalyst quenching by irreversible deprotonation of phenol.

### Screening of acrylate and methacrylate homopolymers

**Fig. 2:**
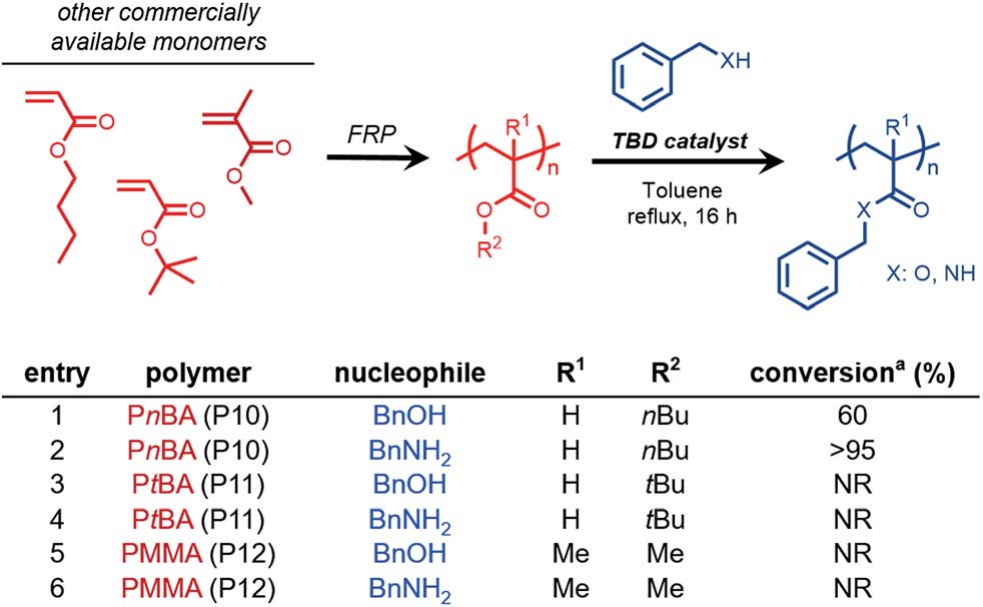
Conventional free-radical polymerization (FRP) and subsequent transesterification and amidation of P*n*BA, P*t*BA, and PMMA with model benzyl nucleophiles. 100 mg mL^–1^ [polymer], [BnXH] : [M] : [TBD] = 4 : 1 : 0.3, NR: no reaction, 1 : 1 v/v DMSO/toluene used for BnNH_2_ (entries 2, 4, and 6) ^a^conversion determined using ^1^H NMR spectroscopy.

We sought to further expand this chemistry to potentially accommodate other commercially relevant acrylic polymers. Conventional free-radical polymerization yielded three acrylic polymers derived from commercially available acrylates and methacrylates, poly(*n*-butyl acrylate) (P*n*BA, **P10**), poly(*tert*-butyl acrylate) (P*t*BA, **P11**), and poly(methyl methacrylate) (PMMA, **P12**). Benzyl alcohol and benzylamine were chosen as model nucleophiles to probe the reactivity (Fig. 2). Transesterification of P*n*BA with benzyl alcohol revealed a mixture of benzyl and *n*-butyl esters, an observation attributed to the inefficient removal of *n*-butanol over the course of the reaction (Fig. 2, entry 1, Fig. S9†).

However, when benzylamine was chosen as the nucleophile, quantitative conversion was achieved (Fig. 2, entry 2, Fig. S10†). Interestingly, when P*t*BA and PMMA were subjected to transesterification and amidation conditions, no conversion was observed even under extended reaction times lasting 48 h. These results were consistent with previous reports involving similar small molecule substrates being unreactive during TBD-catalyzed acyl substitutions.^29^

### Chemoselective transesterification of acrylic copolymers

From these results, we envisioned the chemoselectively afforded by organocatalyzed transesterification could be utilized to develop a new synthetic methodology of chemoselective post-polymerization modification. Specifically, copolymers composed of sterically-differentiated comonomers such as methyl acrylate and methyl methacrylate could serve as substrates for the site-selective installation of functional molecules. Therefore, a series of acrylic and methacrylic copolymers were synthesized to study both chemo- and site-selective functionalization using TBD as an organocatalyst.

Copolymers of methyl acrylate with various methacrylic comonomers were synthesized using free radical polymerization to yield PMMA-*co*-PMA (polymer **CP1**), P*n*BMA-*co*-PMA (**CP2**), PPEGMA-*co*-PMA (**CP3**), and P*t*BA-*co*-PMA (**CP4**).

**Fig. 3:**
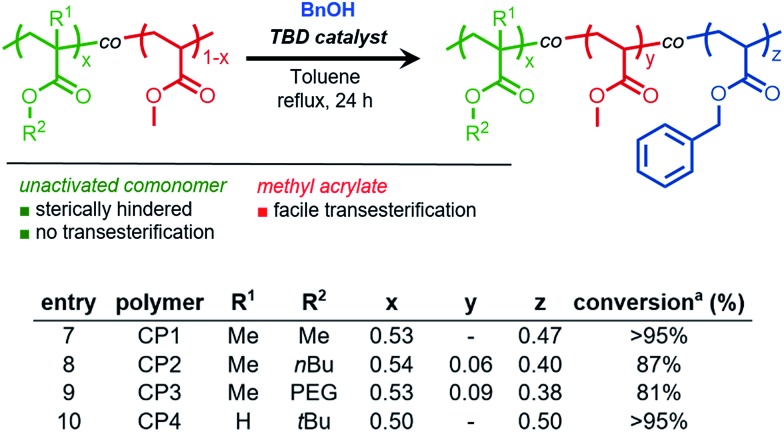
Chemoselective transesterification of acrylic and methacrylic copolymers. 100 mg mL^–1^ [polymer], [benzyl alcohol (BnOH)] : [MA] = 4 : 1 ^a^conversion determined using ^1^H NMR spectroscopy.

BnOH was chosen as a model nucleophile to study the efficiency of methyl acrylate (MA) transesterification in the presence of sterically hindered comonomers. For PMMA-*co*-PMA (Fig. 3, entry 7, Fig. S9†) and P*t*BA-*co*-PMA (Fig. 3, entry 10, Fig. S12†), only the MA units in these copolymers underwent transesterification, clearly demonstrating both the highly efficient and chemoselective nature of TBD for precision functionalization of unhindered acryloyl units in acrylate derived copolymers. However, for acrylate copolymers possessing higher molecular weight primary alkyl side chains, such as those derived from *n*-butyl methacrylate and poly(ethylene glycol) monomethyl ether methacrylate, lower conversion was observed under similar reaction conditions (Fig. S12–S13†). Kinetically, these reactions proceeded at a lower rate at the same mass concentration due to the decreased weight fraction of acrylic methyl esters in the copolymer, thus reducing the molarity of the reaction for a given polymer concentration. Additionally, the lower reactivity in these cases could result from the steric hindrance provided by the adjacent bulky comonomer units surrounding the MA repeat units of **CP2** and **CP3**, which would prevent TBD from efficiently activating the MA esters.

To demonstrate a reasonable application of this functionalization strategy, we designed a method of transforming block copolymers chemoselectively to readily modify the hydrophobic and hydrophilic character of polymer amphiphiles prepared from commercially available monomer feedstocks. A hydrophobic diblock copolymer, namely PMMA-*b*-PMA, was synthesized using RAFT polymerization (*M*_n_ = 28 000 g mol^–1^, *Đ* = 1.29, PMA block = 50 mol%). We then sought to generate an amphiphilic block copolymer product through the addition of diethylene glycol methyl ether (DEG ME) as a hydrophilic alcohol in the presence of TBD. Transesterification of the PMA block with DEG ME achieved high conversion (*ca.* 94%, Fig. S16†), with no functionalization of the MMA block being observed. This example demonstrates the potential of this organocatalyzed reaction strategy being exploited for the chemoselective preparation of a diverse family of block copolymers without the need for specialty activated-ester monomers.

### Chain-end selective transesterification of PMMA

Finally, to further illustrate the highly selective nature of TBD-catalyzed transesterification, we designed a synthetic strategy for achieving chain-end selective transesterification of PMMA. RAFT polymerization was employed to synthesize PMMA in the presence of a dithiobenzoate chain-transfer agent (*M*_n GPC MALS_ = 5400 g mol^–1^, *Đ* = 1.06, Fig. S17†).

**Fig. 4:**
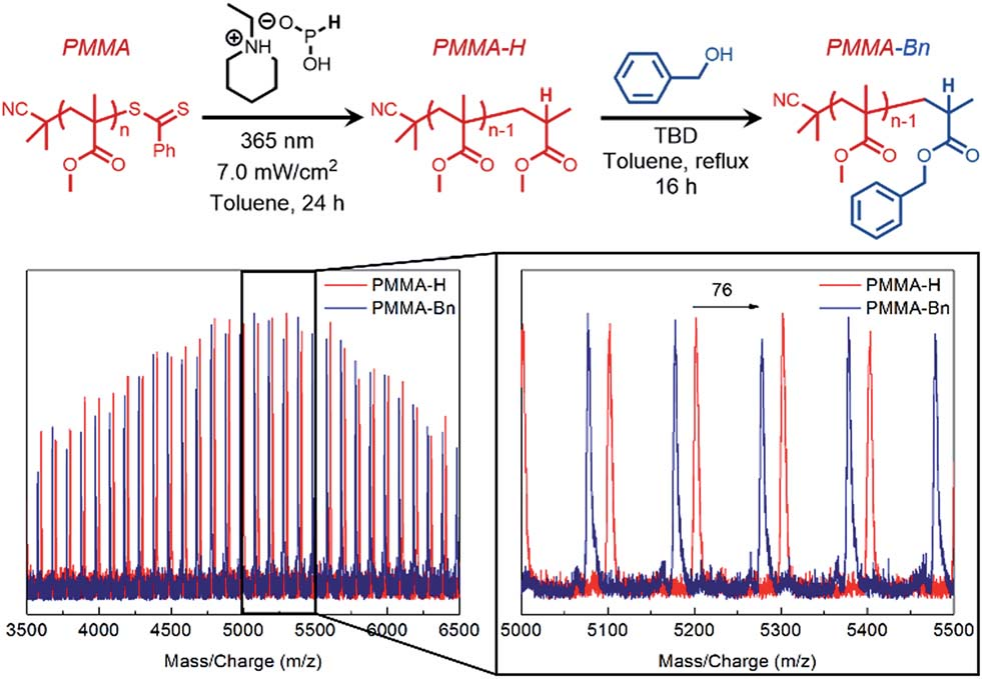
Matrix assisted laser desorption/ionization time-of-flight mass spectrometry (MALDI-TOF-MS) spectrum of chain-end selective transesterification of end-group removed PMMA (PPMA-H, red) resulting in terminal functionalized PPMA (PPMA-Bn, blue).

To sterically access the ω chain-end for catalytic activation, a photoinduced end-group removal strategy^30^ previously developed in our group was used as the key step in generating a hydrogen capped PMMA (PMMA-H) that contained a sterically unhindered ester on the ω terminus. Transesterification of PMMA-H resulted in the monosubstituted ω-functionalized product (PMMA-Bn) which was verified through MALDI-TOF-MS (Fig. 4) that showed a shift of 76 g mol^–1^, which matches the theoretical shift in molecular weight.

This “pin-point” functionalization method of a polymer that would typically be considered relatively non-reactive further demonstrates the promise of TBD-catalyzed derivatization of polyacrylate side chains or polymethacrylate end groups.

## Conclusions

In conclusion, we have developed a strategy for the catalytic activation and subsequent functionalization of polyacrylates through the use of TBD as a nucleophilic catalyst. The selective removal of methanol formed during poly(methyl acrylate) transesterification allowed for quantitative functionalization and proved to be a suitable strategy for the efficient installation of functional alcohols. As compared to approaches that involve functional precursors that contain activated esters, the strategy reported here relies on polyacrylates that effectively have extended shelf-lives due to their hydrolytic stability. Furthermore, this method provided access to site-selective polymer functionalization through chemoselective transesterification of block copolymers, statistical copolymers, as well as homopolymers.

A variety of synthetic applications can be envisioned utilizing this methodology such as the chemical upcycling of adhesives and coatings waste into value added materials. Such methodology developments are of growing importance due to issues concerning the long-term limitations of materials derived from nonrenewable resources. In addition to chemical repurposing of waste materials, this strategy can be used as a new synthetic tool in the chemoselective activation of polymeric materials to precisely functionalize well-defined macromolecules.

## Conflicts of interest

There are no conflicts to declare.

## Supplementary Material

Supplementary information
