# Supplementary material for: Synthetic upcycling of polyacrylates through organocatalyzed post-polymerization modification
Source: Chem Sci. 2017 Sep 29;8(11):7705–9. doi: 10.1039/c7sc02574b (PMC5851076; doi:10.1039/c7sc02574b)
Supplement: Supplementary file 1 [file SC-008-C7SC02574B-s001.pdf]

Electronic Supplementary Material (ESI) for Chemical Science.
This journal is © The Royal Society of Chemistry 2017

Synthetic Upcycling of Polyacrylates through Organocatalyzed Post-Polymerization Modification

Charles P. Easterling, Tomohiro Kubo, Zachary M. Orr, Gail E. Fanucci*, Brent S. Sumerlin*

George & Josephine Butler Polymer Research Laboratory, Center for Macromolecular Science & Engineering, Department of Chemistry, University of Florida, PO Box 117200, Gainesville, Florida 32611-7200, United States

Table of Contents

General Experimental **2–6**
- Materials **2**
- Instrumentation **2-3**
- RAFT polymerization of MA **3**
- Synthesis of **P10-P12** **3**
- Synthesis of **CP1-CP4** **3-4**
- Synthesis of PMMA RAFT Macro-CTA **4**
- Synthesis of PMMA-*b*-PMA **5**
- General procedure for TBD-catalyzed transesterification of polyacrylates **5**
- Synthesis of end-group removed PMMA **5-6**
- Chain-end selective transesterification of PMMA **6**

Figure S1. GPC chromatogram of PMA homopolymer **7**
Figure S2. ^{1}H NMR spectrum of **P1** **8**
Figure S3. ^{1}H NMR spectrum of **P2** **9**
Figure S4. ^{1}H NMR spectrum of **P3** **10**
Figure S5. ^{1}H NMR spectrum of **P4** **11**
Figure S6. ^{1}H NMR spectrum of **P5** **12**
Figure S7. ^{1}H NMR spectrum of **P6** **13**
Figure S8. ^{1}H NMR spectrum of **P7** **14**
Figure S9. ^{1}H NMR spectrum of **P10** transesterification product **15**
Figure S10. ^{1}H NMR spectrum of **P10** amidation product **16**
Figure S11. ^{1}H NMR spectrum of **CP1** **17**
Figure S12. ^{1}H NMR spectrum of **CP2** **18**
Figure S13. ^{1}H NMR spectrum of **CP3** **19**
Figure S14. ^{1}H NMR spectrum of **CP4** **20**
Figure S15. ^{1}H NMR spectrum of PMMA-*b*-PMA **21**
Figure S16. ^{1}H NMR spectrum of PMMA-*b*-PDEGA **22**
Figure S17. GPC chromatogram of PMMA homopolymer **23**
References **23**

General Experimental

Materials

2-(Dodecylthiocarbonothioylthio)-2-methylpropionic acid (DMP),[1] 4-cyano-4-[(dodecylsulfanylthiocarbonyl)sulfanyl]petanoic acid, 2-cyano-2-propyl benzodithioate (CPD)[2], and *N*-boc-ethanolamine[3] were synthesized according to previous reports. Benzylamine ($BnNH_2$, Sigma-Aldrich, 99%) was distilled prior to use. Benzyl alcohol (BnOH, Alfa Aesar, 99%), furfuryl alcohol (Alfa Aesar, 98%), cinnamyl alcohol (Sigma-Aldrich, 98%), 9-anthracenemethanol (Sigma Aldrich, 97%), cyclohexanol (Sigma-Aldrich, 99%), tert-butyl alcohol (Fisher, >99%), phenol (Sigma-Aldrich, >99%), diethylene glycol methyl ether (DEG, Sigma-Aldrich, 99%), and 1-ethylpiperidine hypophosphite (EPHP, Sigma-Aldrich, 95%) were all used as received. Methyl methacrylate (MMA, Sigma-Aldrich, 99%), methyl acrylate (MA, Alpha Aesar, 99%), *n*-butyl acrylate (*n*BA, Alpha Aesar, 99%), *t*-butyl acrylate (*t*BA, Sigma-Aldrich, 99%), and poly(ethylene glycol) monomethyl ether methacrylate (PEGMA, M_n = 500 g/mol, Sigma-Aldrich, 99%) were passed through basic alumina to remove acidic impurities. 2,2'-Azobis(isobutyronitrile) (AIBN, Sigma-Aldrich, 98%) was recrystallized from ethanol. Toluene (Sigma Aldrich, 99%) and DMSO (VWR) were stored over 4 Å molecular sieves for 24 h prior to use.

Instrumentation

Nuclear Magnetic Resonance (NMR) Spectroscopy. ^1H NMR spectra were recorded on a Varian Innova2 500 MHz NMR spectrometer using the residual solvent signal as a reference.

Size Exclusion Chromatography (SEC). Molecular weights and molecular weight distributions were determined *via* multi-angle laser light scattering size exclusion chromatography (MALS-SEC) in *N,N*-dimethylacetamide (DMAc) with 50 mM LiCl at 50 °C and a flow rate of 1.0 mL/min (Agilent isocratic pump, degasser, and autosampler; ViscoGel I-series 10 μm guard column and two ViscoGel I-series G3078 mixed bed columns, with molecular weight ranges $0-20\times10^3$ and $0-10\times10^6$ g/mol, respectively). Detection consisted of a Wyatt Optilab T-rEX refractive index detector operating at 658 nm and a Wyatt miniDAWN Treos light scattering detector operating at 659 nm. Absolute molecular weights and polydispersities were calculated using Wyatt ASTRA software.

Matrix assisted laser desorption ionization (MALDI) mass spectrometry. Matrix assisted laser desorption/ionization time-of-flight (MALDI-TOF/TOF) was performed on a Bruker Microflex LRF MALDI TOF (Billerica, MA) mass spectrometer in reflectron, positive ion mode using an N2 onaxis laser. Spectra were collected in flexControl (Bruker Daltronics Inc., Billerica, MA) and analyzed using flexAnalysis (Bruker Daltronics Inc., Billerica, MA) and Polymerix Version 3 software (Sierra Analytics, Modesto, CA).

RAFT polymerization of PMA

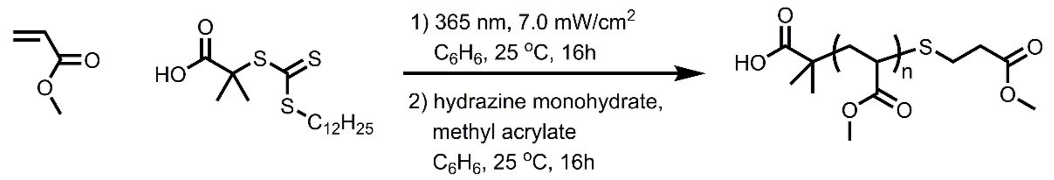

Methyl acrylate (5.0 g, 58 mmol) and 2-(dodecylthiocarbonothioylthio)-2-methylpropionic acid (210 mg, 0.58 mmol) were dissolved in 14.5 mL of benzene and transferred to a 25 mL Schlenk tube. The solution was purged under argon for 20 min with stirring. The reaction vessel was positioned 3 cm above a commercially available UV light source having a light intensity of 7.0 mW/cm^2 and allowed to irradiate at room temperature for 16 h. Hydrazine monohydrate (140 µL, 2.9 mmol) was then added and allowed to stir for 3 h at room temperature. MA (2.5 g, 29 mmol) was then added and allowed to stir for an additional 13 h. The product was purified by dialysis (Spectra/Por 3.5 kD MWCO) in acetone followed by drying under reduced pressure. ($M_{n\ GPC\ MALS}$ = 9,800 g/mol, M_w/M_n = 1.20)

*General synthesis of **P8-P10**: Conventional Free-Radical Polymerization*

Acrylate and methacrylate monomers (*n*-butyl acrylate (*n*BA), *t*-butyl acrylate (*t*BA), and methyl methacrylate (MMA)) were polymerized on a 5 gram scale using conventional radical polymerization in the presence of 1 mol% AIBN. Toluene was used as a universal polymerization solvent, with the exception of P*t*BA which used anisole as the solvent. The polymerizations were carried out in a preheated oil bath set at 70 °C for 16 h with an initial monomer concentration of 2 M. Polymers P8-P10 were then purified by rotary evaporation to remove residual monomer and solvent, followed by dialysis (Spectra/Por 3.5 kD MWCO) against acetone. Final products were

obtained upon removal of acetone using rotary evaporation and further drying under reduced pressure.

Synthesis of ***CP1 – CP4****: Conventional Free-Radical Polymerization*

Methyl acrylate (1.0 eq.) and methacrylic or acrylic comonomer (1.0 eq. *n*-BMA, MMA, *t*-BA, or PEGMA) were dissolved in toluene at an initial monomer concentration of 2 M. AIBN (1.0 mol% relative to monomer) was then added, and the solution was allowed to purge under argon for 20 minutes prior to the reaction. The reaction was carried out in a preheated oil bath set at 70 °C for 16 h. Polymers CP1-CP4 were then purified by rotary evaporation to remove residual monomer and solvent, followed by dialysis (Spectra/Por 3.5 kD MWCO) in acetone. Final products were obtained upon removal of acetone using rotary evaporation and further drying under reduced pressure.

Synthesis of PMMA RAFT Macro-CTA

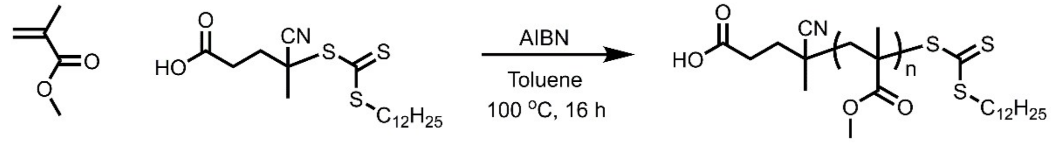

Methyl methacrylate (5.00 g, 49.9 mmol), 4-cyano-4-[(dodecylsulfanylthiocarbonyl)sulfanyl]-petanoic acid (134 mg, 3.32 × 10^{-1} mmol), and AIBN (5.50 mg, 3.35 × 10^{-2} mmol) were dissolved in 5 mL of toluene and transferred to a 10 mL schlenk tube. The solution was then degassed under argon while stirring continuously for 20 min prior to submerging in a preheated oil bath set to 100 °C. After 16 h the polymer was precipitated twice into cold methanol to afford 3.18 g of PMMA macro-CTA ($M_{\mathrm{n\ GPC\ MALS}}$ = 12,500 g/mol, M_w/M_n = 1.18).

Synthesis of PMMA-b-PMA via RAFT polymerization

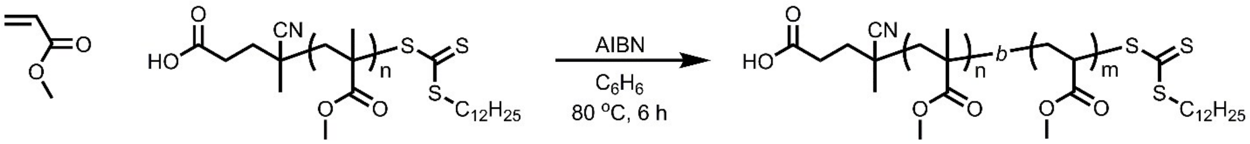

Methyl acrylate (MA, 1.03 g, 12.0 mmol), previously synthesized PMMA macro-CTA (1.00 g, 8.00×10^{-2} mmol), and AIBN (1.30 mg, 7.92×10^{-2} mmol) were dissolved in 9 mL of benzene and transferred to a 10 mL schlenk tube. The solution was then degassed under argon while stirring continuously for 20 min prior to submerging in a preheated oil bath set to 80 °C. After 6 h the polymer was precipitated twice into hexanes to afford 1.38 g of $PMMA_{125}$-*b*-PMA_{122} (conversion = 92%, $M_{\mathrm{n\ theor}}$ = 24,380 g/mol $M_{\mathrm{n\ GPC\ MALS}}$ = 23,000 g/mol, M_w/M_n = 1.29).

General procedure for TBD-catalyzed transesterification of polyacrylates

All reactions were carried out in a fume hood using flame-dried glassware. Previously synthesized polymer (1.0 eq. methyl esters) and nucleophile (1.5 eq.) were added to a 10 mL round bottom flask and dissolved in toluene at a polymer concentration of 100 mg/mL. Triazabicyclodecene (15 mol%) was then added and the flask was equipped with a short reflux condenser and allowed to purge under argon for 20 min. Reactions were carried under reflux conditions in a preheated oil bath set at 120 ºC for 16 h under constant, gentle argon flow to remove methanol over the course of the reaction. In the case of benzylamine, a 50/50 v/v toluene/DMSO solvent system was used. The resultant modification reactions were monitored by ^{1}H NMR spectroscopy by withdrawing 100 µL aliquots from the reaction solution. After the reaction, the solution was cooled to room temperature and diluted with dicholoromethane (100 mL). TBD was then removed by washing the organic layer with 0.1 M HCl (5 x 50 mL). The organic layer was then dried with anhydrous magnesium sulfate prior to removal of dichloromethane using rotary evaporation, followed by further drying under reduced pressure.

Synthesis of end-group removed PMMA

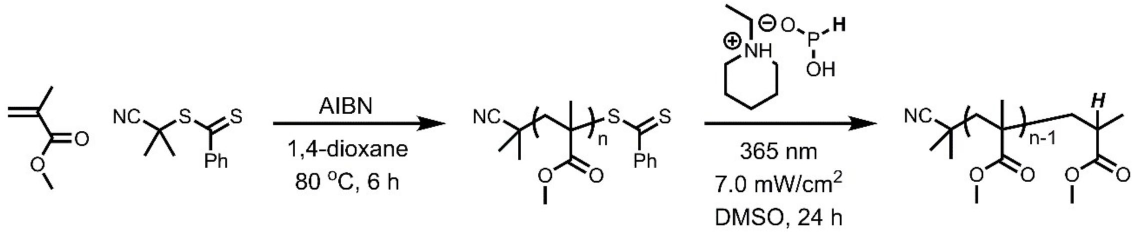

MMA (5.0 g, 5.0 × 10^1 mmol), 2-cyano-2-propyl benzodithioate (550 mg, 2.5 mmol), and AIBN (8.0 mg, 4.9 × 10^{-2} mmol) were dissolved in 12.5 mL of 1,4-dioxane and transferred to a 25 mL Schlenk tube. The solution was then degassed under argon while stirring continuously for 20 min prior to submerging in a preheated oil bath set to 80 °C. After 6 h the polymer was precipitated into hexanes to afford PMMA (M_n = 5,700 g/mol, M_w/M_n = 1.06). The dithiobenzoate end-group was then removed using photoinduced end-group removal with EPHP as reported previously.[4] The product was purified by dialysis (Spectra/Por 3.5 kD MWCO) in acetone followed by drying under reduced pressure.

Chain-end selective transesterification of PMMA

End-group removed PMMA (0.5 × 10^2 mg, 0.5 mmol methyl esters), triazabicyclodecene (7.0 × 10^1 mg, 0.5 × 10^{-2} mmol), and benzyl alcohol (8.0 mg, 0.75 mmol) were dissolved in 0.5 mL of toluene and transferred to a 5 mL round bottom flask equipped with a reflux condenser. The solution was then allowed to purge under argon for 20 min prior to submerging in a preheated oil bath set at 120 ºC for 16 h under constant argon flow. After the reaction, the solution was cooled to room temperature and diluted with dicholoromethane (10 mL). TBD was then removed by washing the organic layer with 0.1 M HCl (5 x 10 mL). The organic layer was then dried with anhydrous magnesium sulfate prior to removal of dichloromethane using rotary evaporation. The product was further purified by dialysis (Spectra/Por 1 kD MWCO) in acetone followed by drying under reduced pressure.

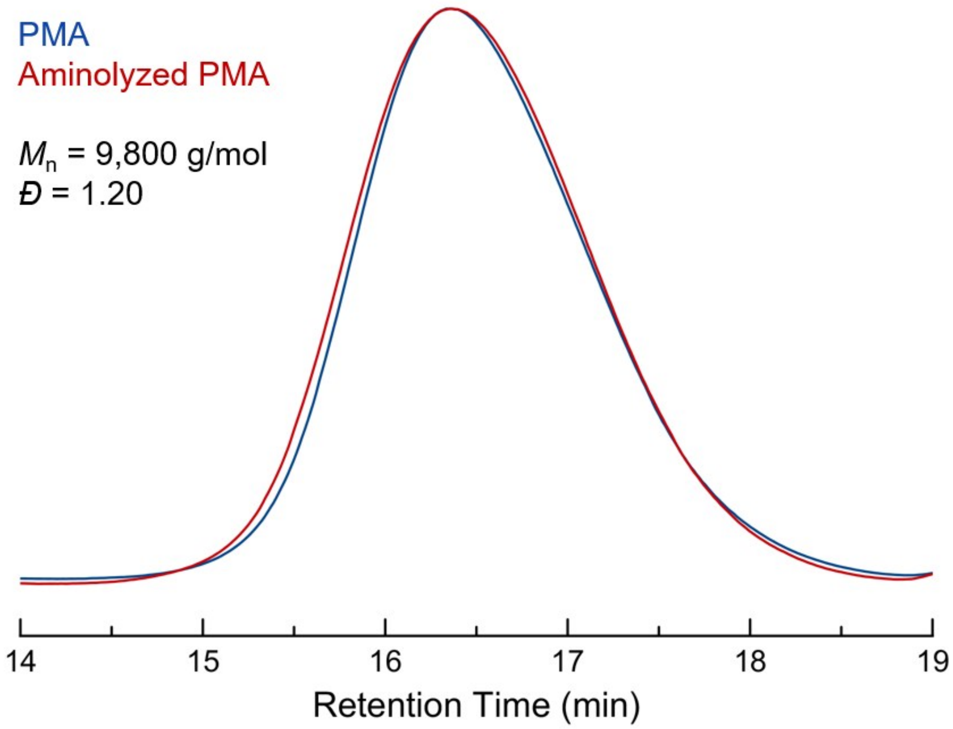

Figure S1. SEC chromatogram of PMA homopolymer.

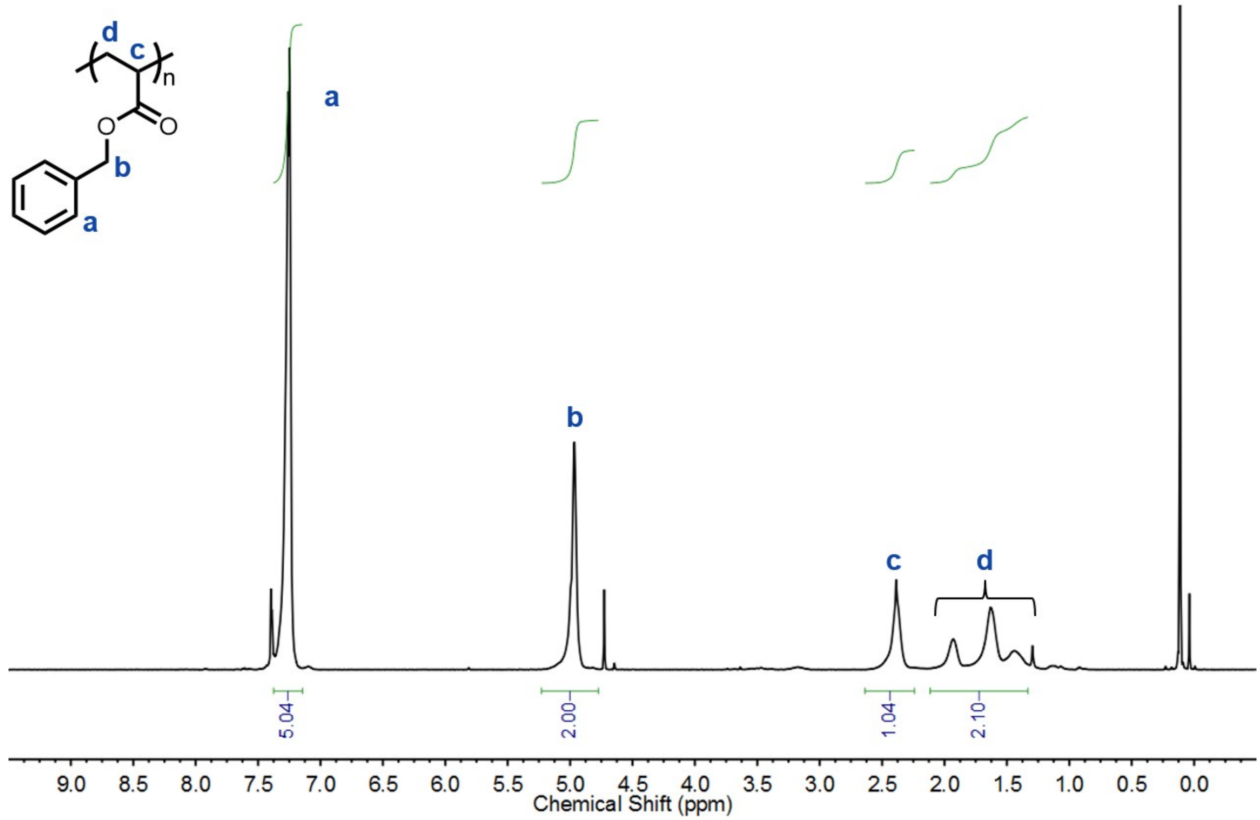

Figure S2. ^{1}H NMR spectrum of **P1** in $CDCl_3$ at 25 °C.

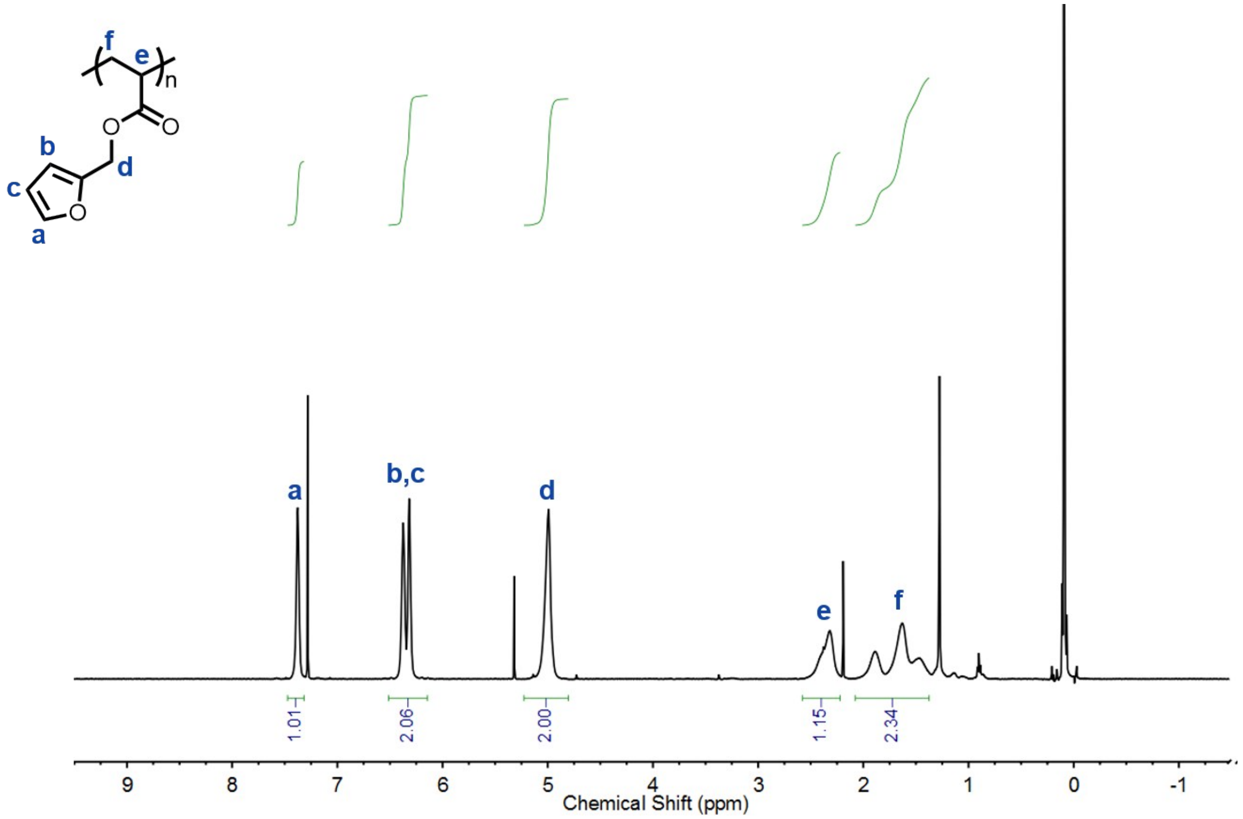

Figure S3. ^{1}H NMR spectrum of **P2** in $CDCl_3$ at 25 °C.

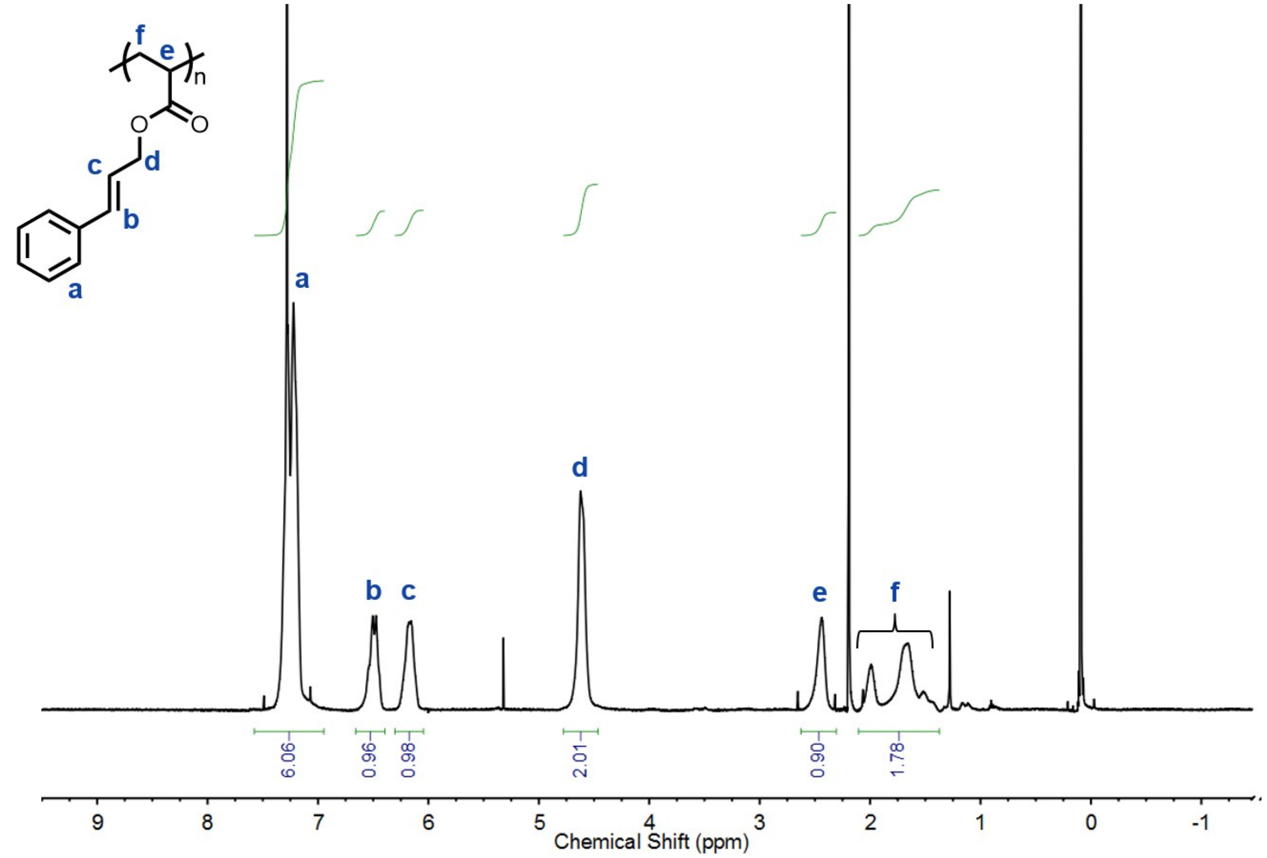

Figure S4. ^{1}H NMR spectrum of **P3** in $CDCl_3$ at 25 °C.

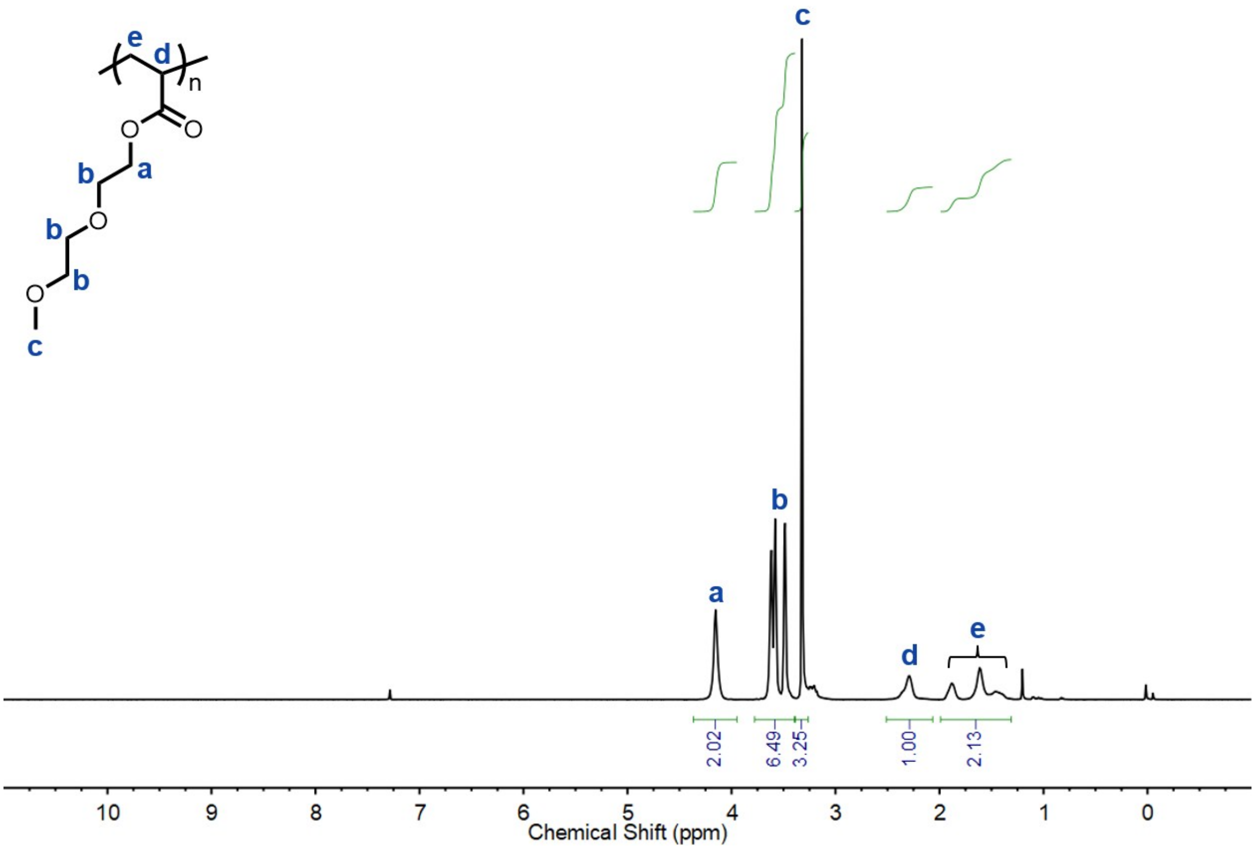

Figure S5. ¹H NMR spectrum of **P4** in $CDCl_3$ at 25 °C.

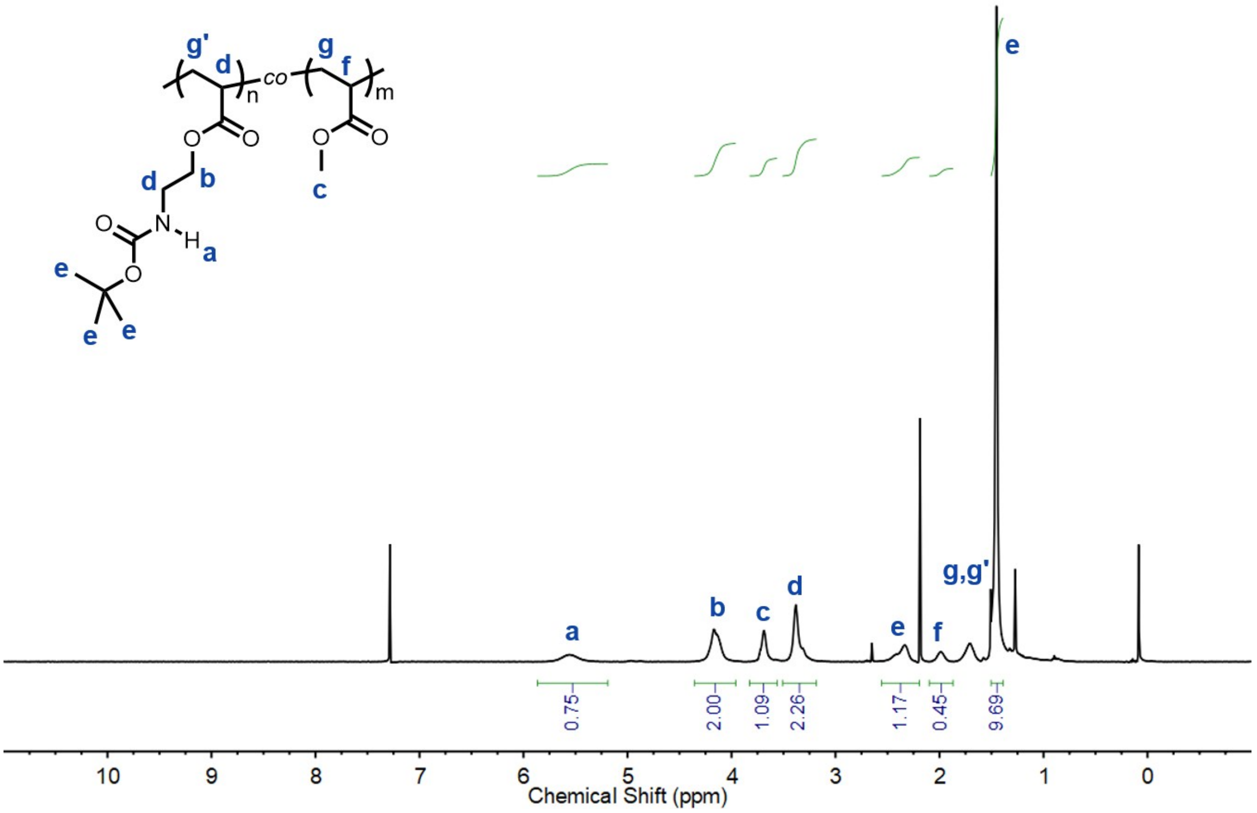

Figure S6. ^{1}H NMR spectrum of **P5** in $CDCl_3$ at 25 °C.

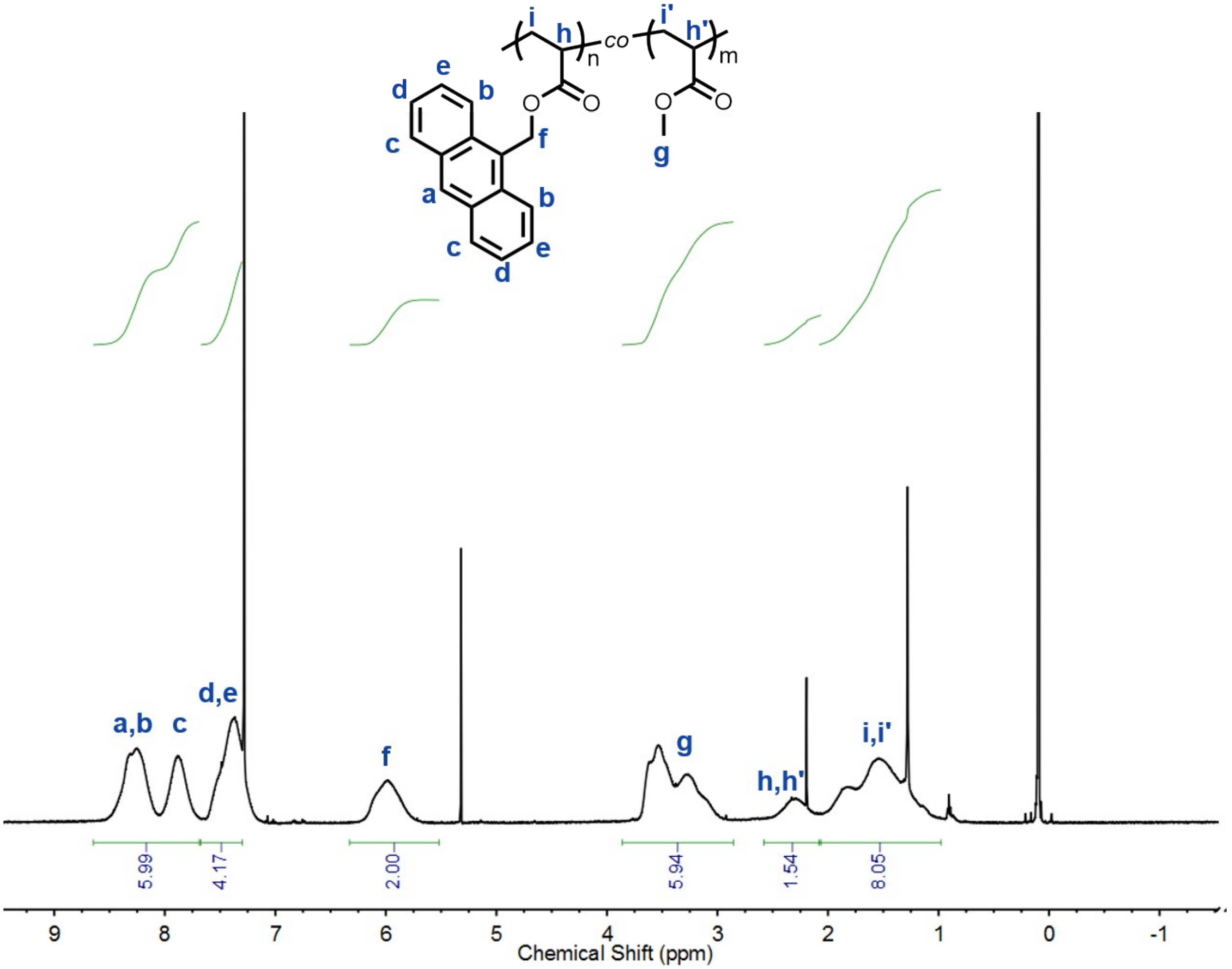

Figure S7. ^{1}H NMR spectrum of **P6** in $CDCl_3$ at 25 °C.

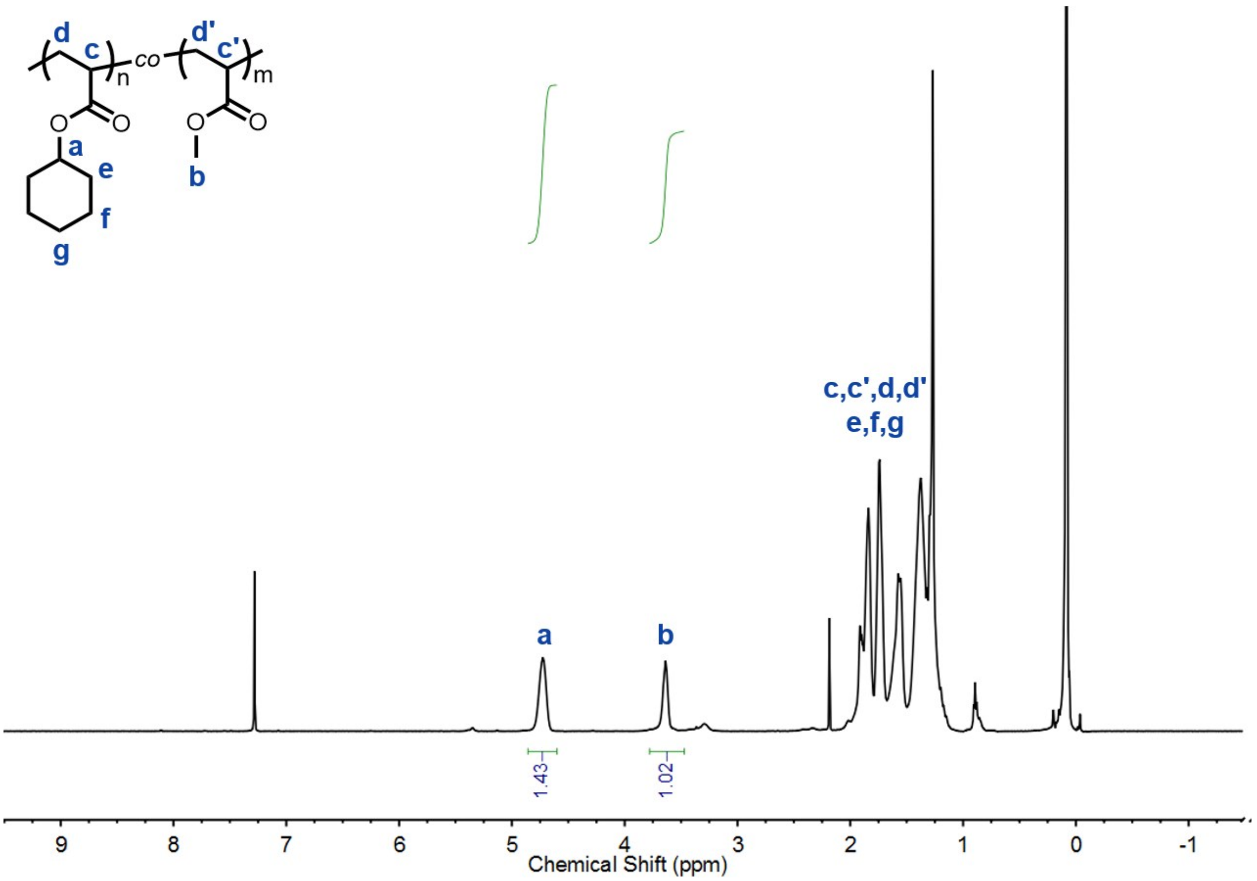

Figure S8. ^{1}H NMR spectrum of **P7** in $CDCl_3$ at 25 °C.

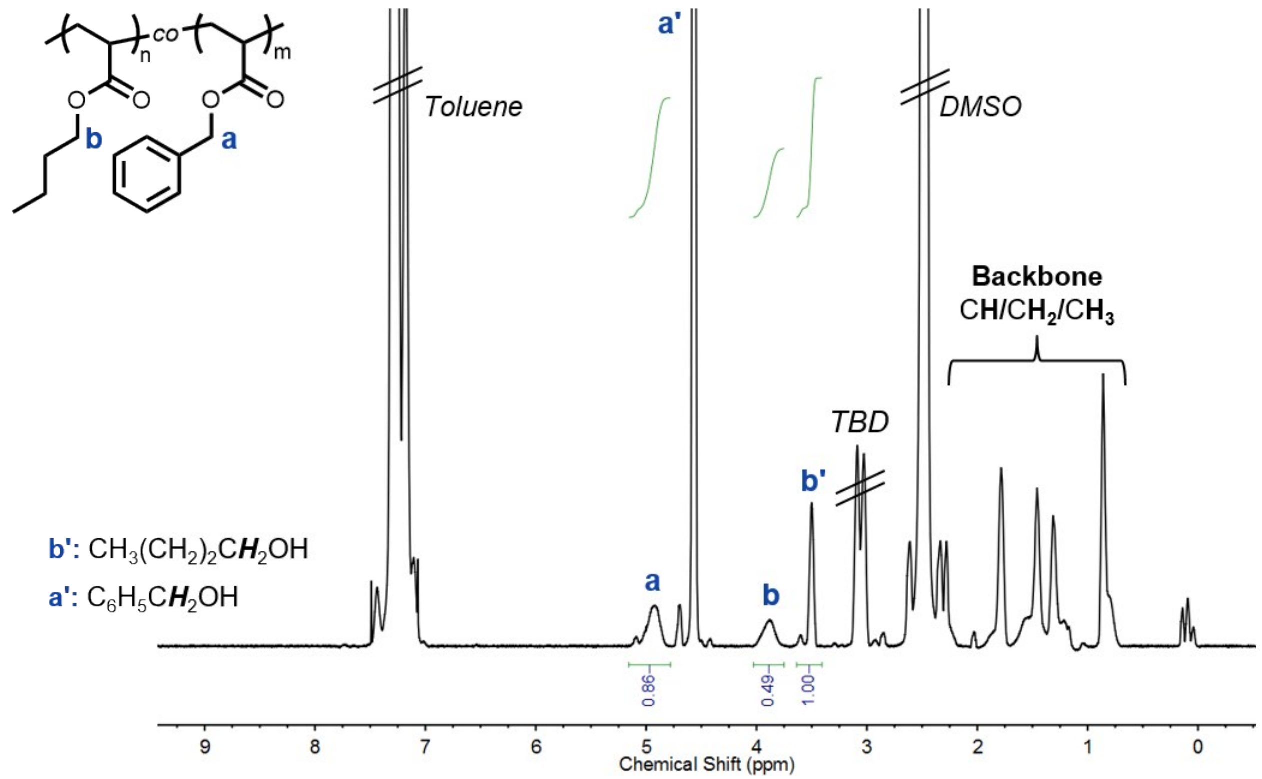

Figure S9. ^{1}H NMR spectrum of a kinetic aliquot of **P10** upon attempted transesterification with benzyl alcohol.

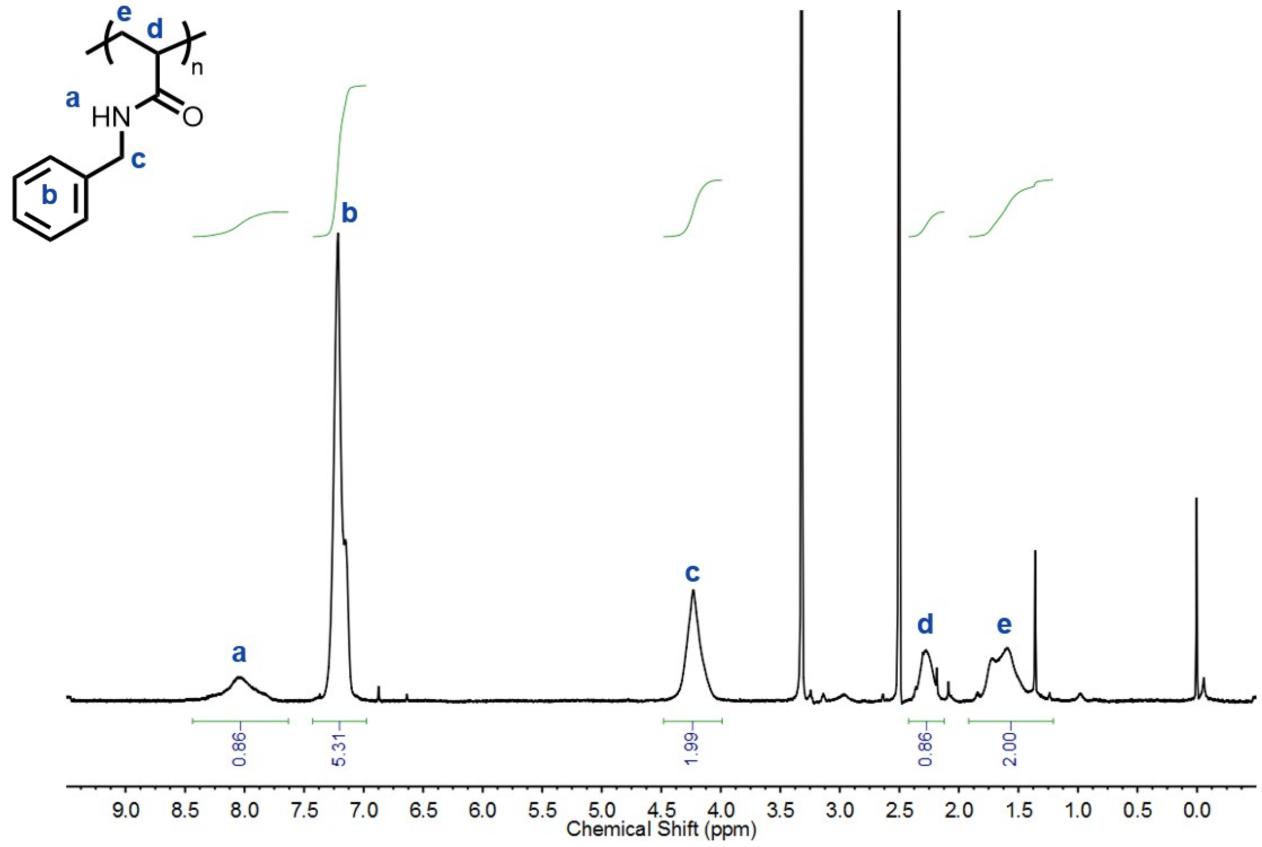

Figure S10. ^{1}H NMR spectrum of **P10** upon amidation with benzyl amine.

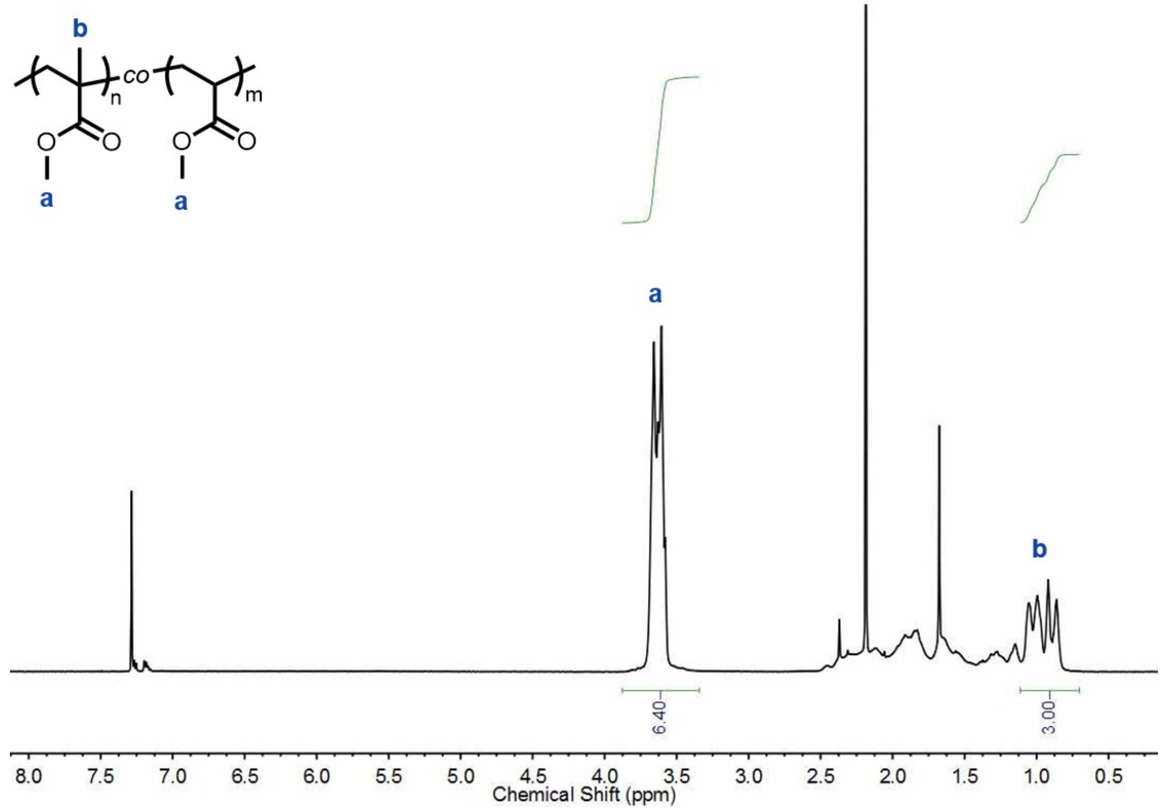

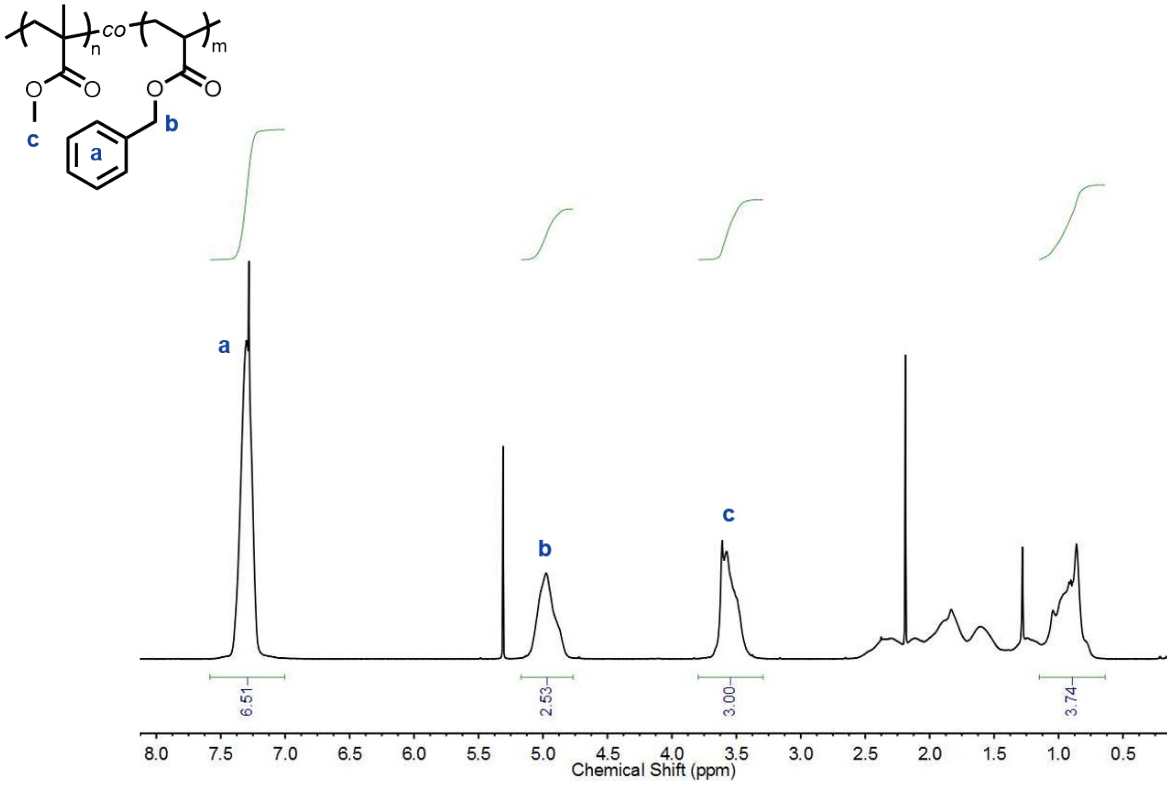

Figure S11. ^{1}H NMR spectra of PMMA-*co*-PMA substrate (top) and product **CP1** after transesterification with benzyl alcohol (bottom).

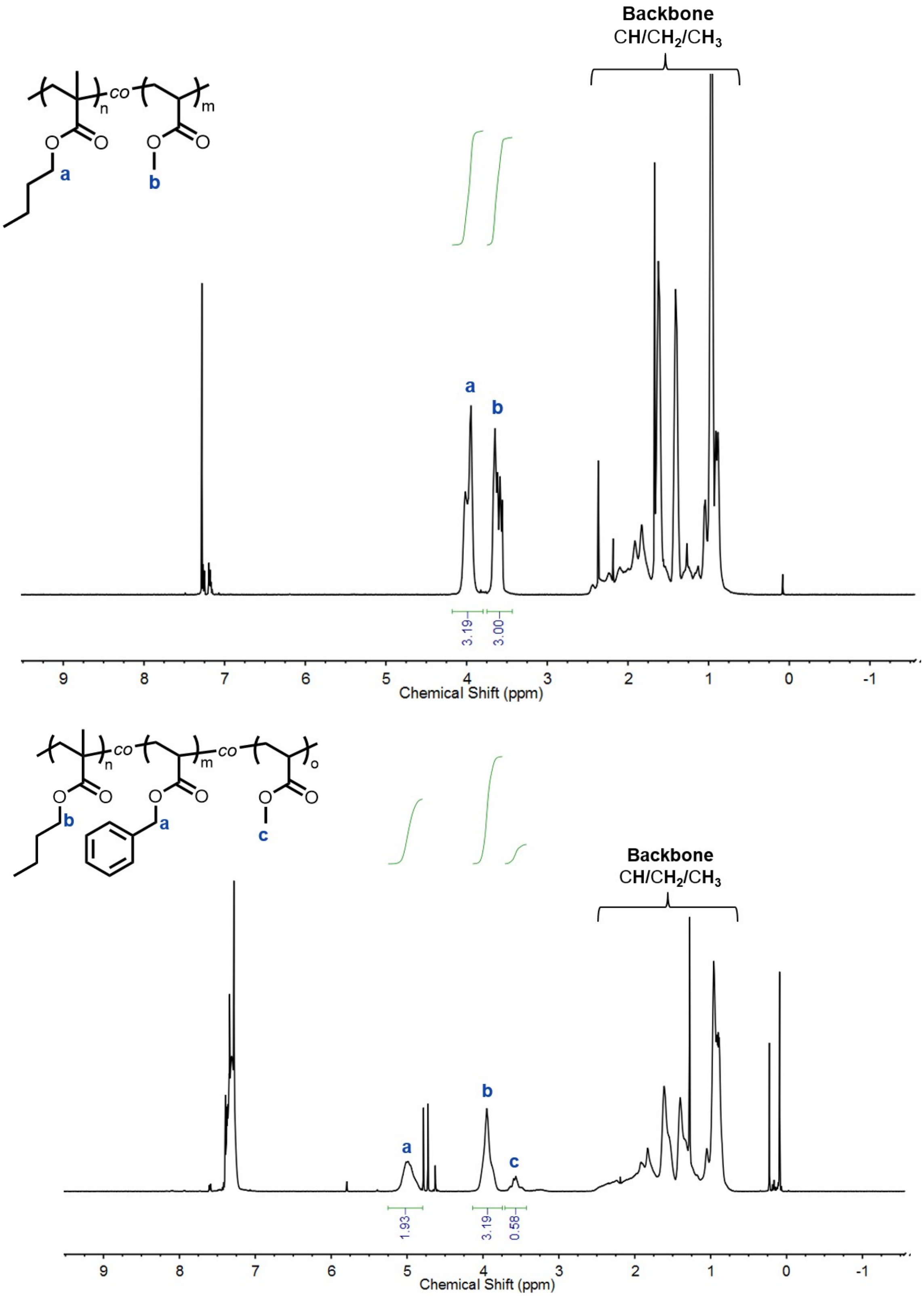

Figure S12. ^{1}H NMR spectra of P*n*BMA-*co*-PMA substrate (top) and product **CP2** after transesterification with benzyl alcohol (bottom).

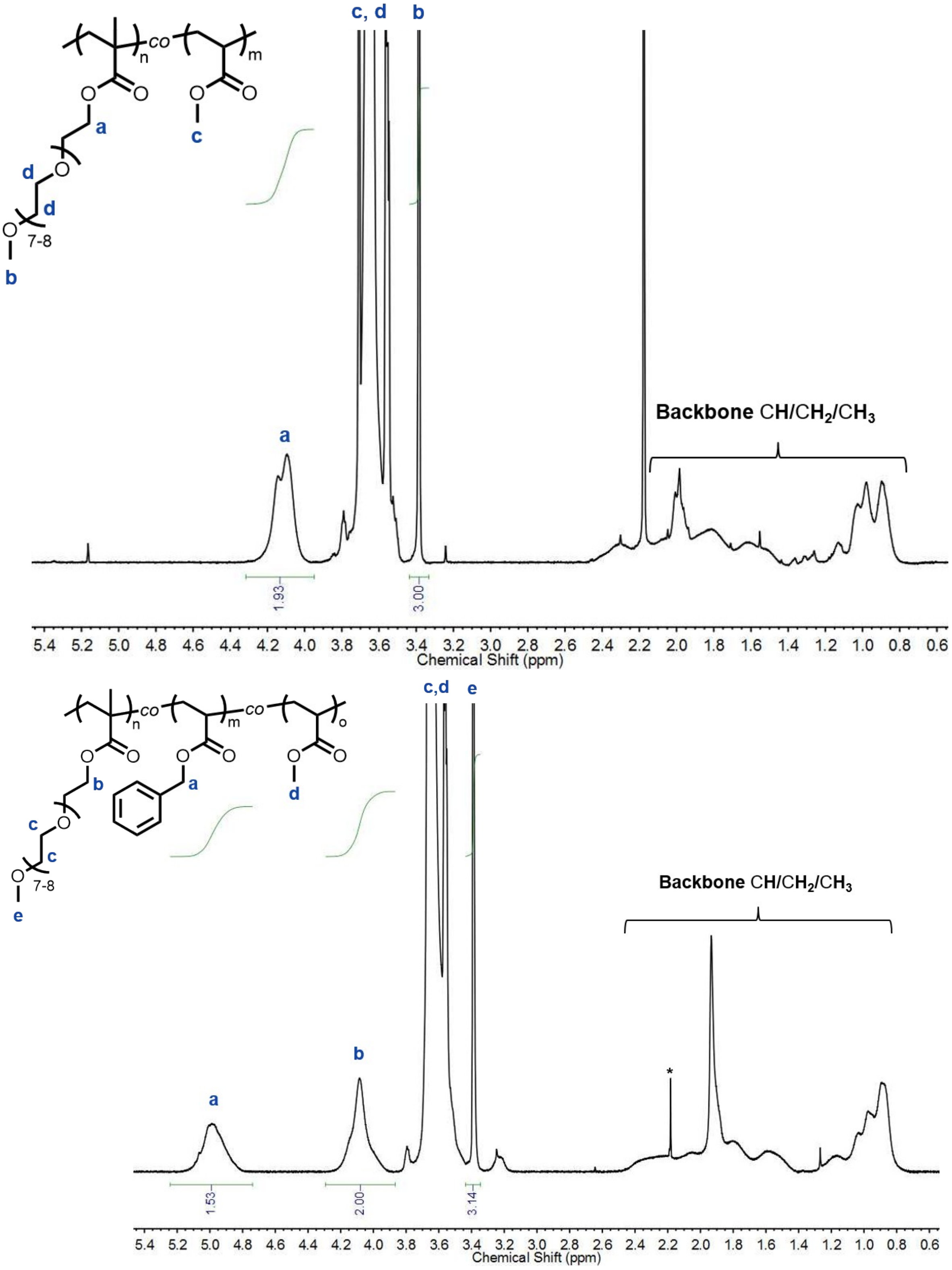

Figure S13. ^{1}H NMR spectra of PPEGMA-*co*-PMA substrate (top) and product **CP3** after transesterification with benzyl alcohol (bottom).

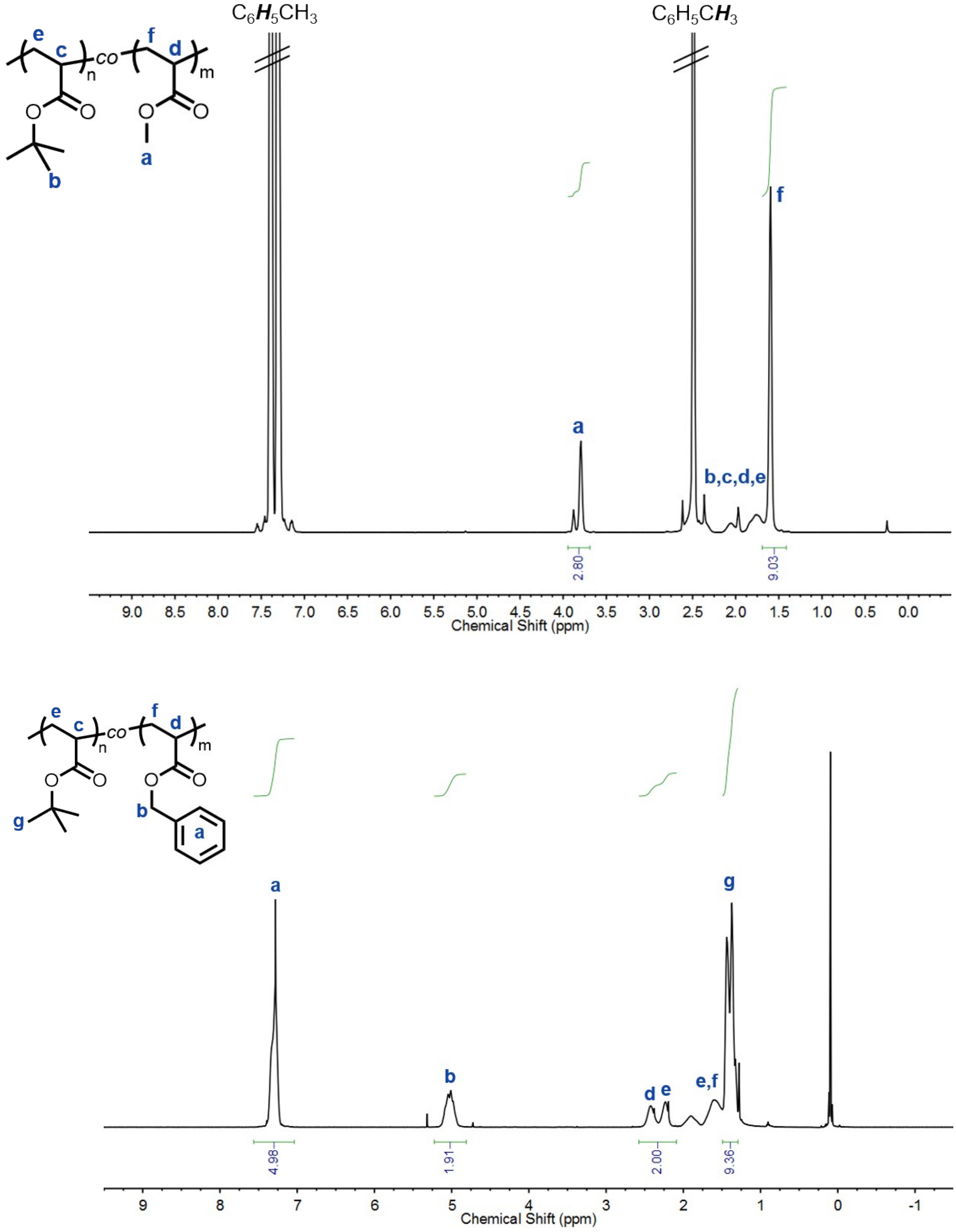

Figure S14. ^{1}H NMR spectra of PtBA-*co*-PMA substrate (top) and product **CP4** after transesterification with benzyl alcohol (bottom)

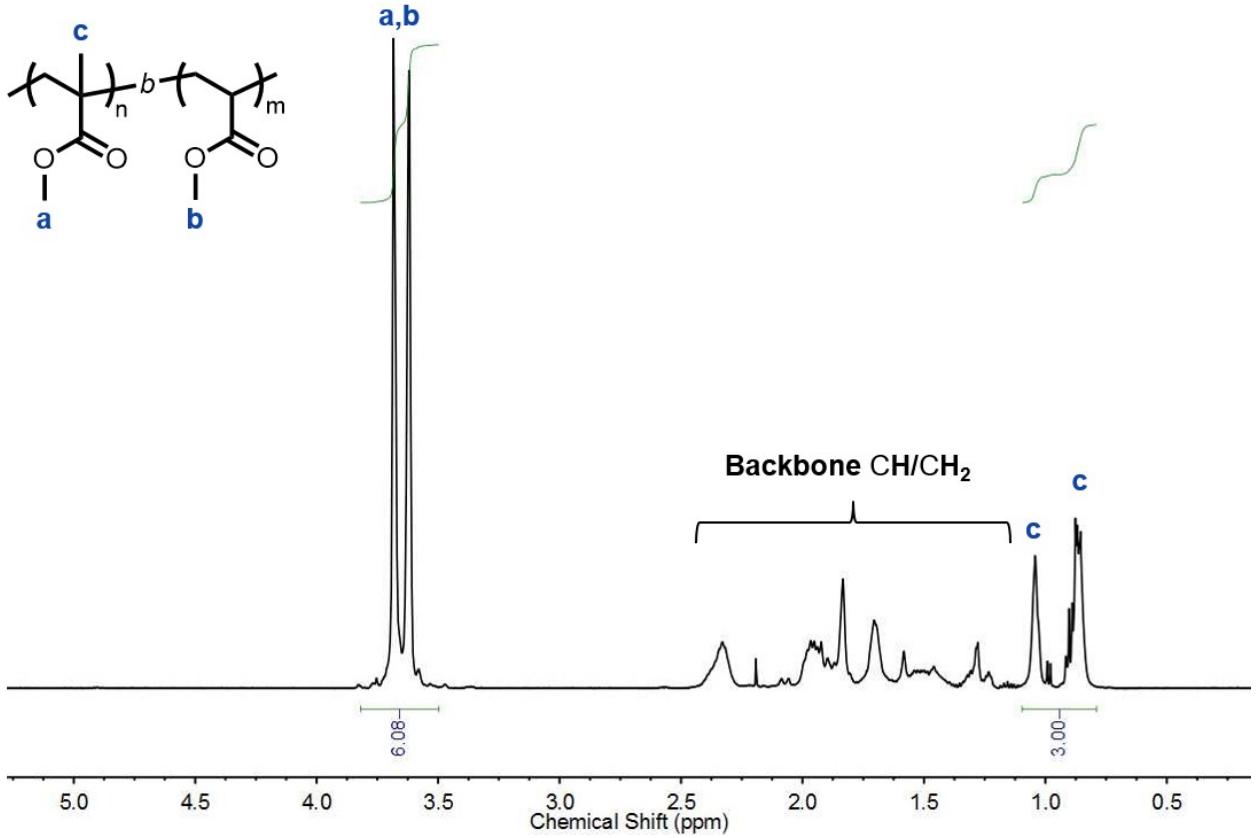

Figure S15. ^{1}H NMR spectrum of PMMA$_{125}$-*b*-PMA$_{122}$ substrate

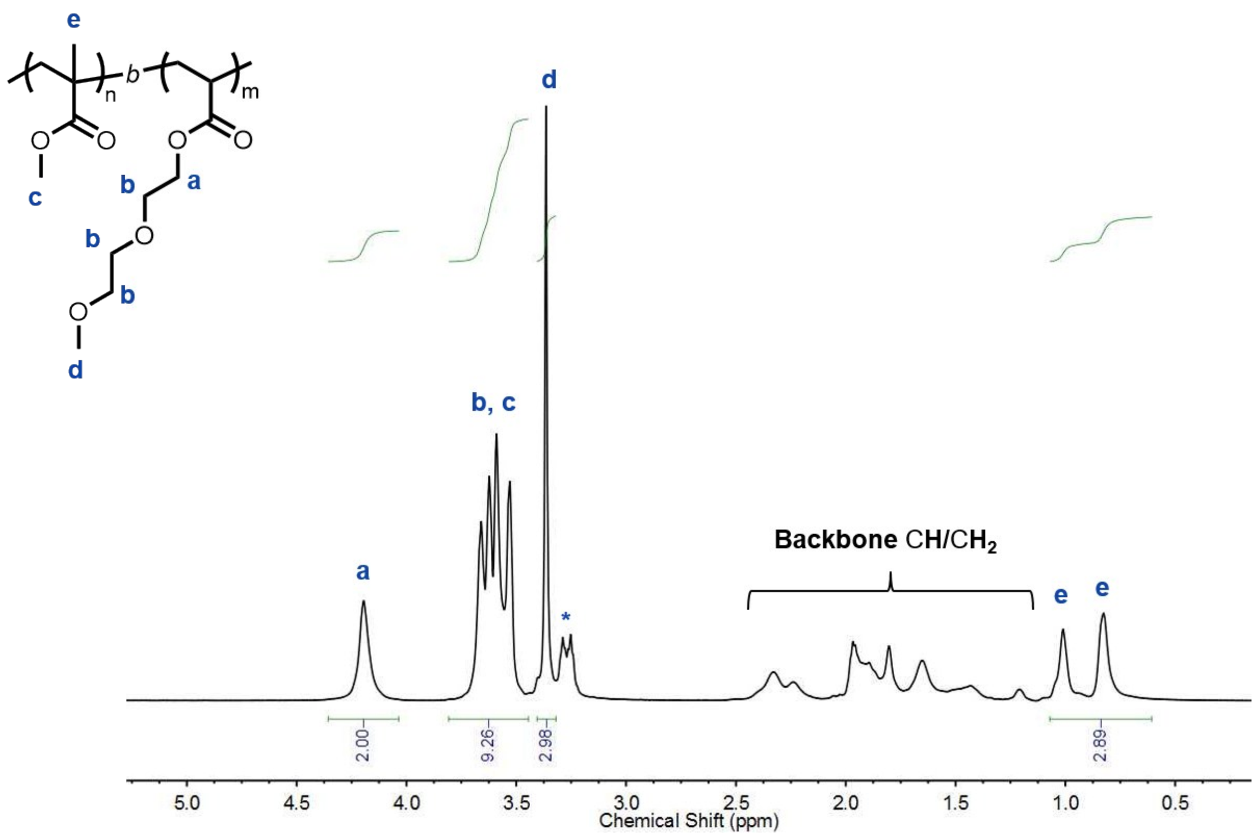

Figure S16. ^{1}H NMR spectrum of PMMA$_{125}$-*b*-PDEGA$_{122}$ transesterification product

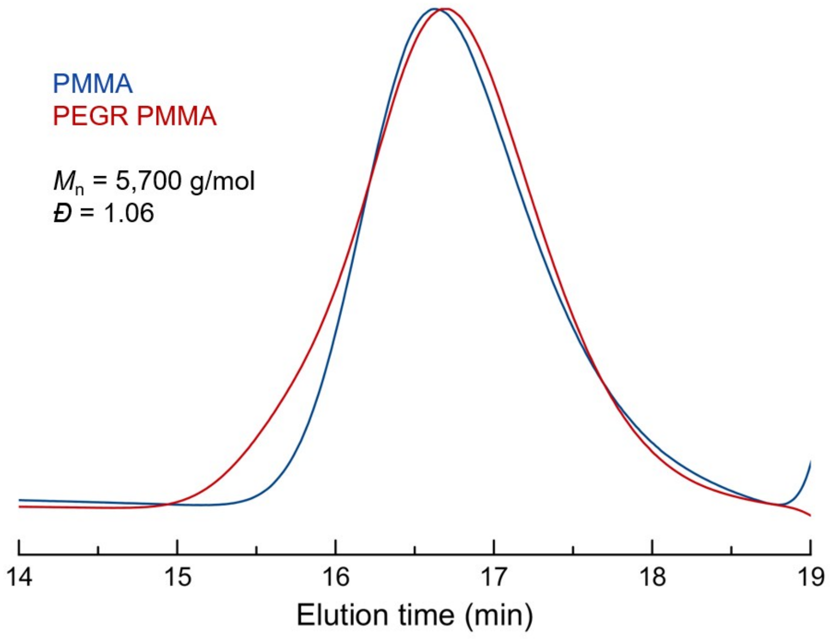

Figure S17. SEC chromatogram of dithiobenzoate terminated PMMA (blue) and hydrogen capped PMMA (red) after photoinduced end-group removal

References

1. J. T. Lai, D. Filla and R. Shea, *Macromolecules*, 2002, **35**, 6754-6756.
2. S. H. Thang, Y. K. Chong, R. T. A. Mayadunne, G. Moad and E. Rizzardo, *Tetrahedron Letters*, 1999, **40**, 2435-2438.
3. G. Helmchen, J.-M. Becht and O. Meyer, *Synthesis*, 2003, **18**, 2805-2810.
4. R. N. Carmean, C. A. Figg, G. M. Scheutz, T. Kubo and B. S. Sumerlin, *ACS Macro Lett.*, 2017, **6**, 185-189.
